# Transmission of SARS-CoV-2 during indoor clubbing events: A clustered randomized, controlled, multicentre trial protocol

**DOI:** 10.3389/fpubh.2022.981213

**Published:** 2022-11-04

**Authors:** Jeanne Goupil de Bouillé, Liem Binh Luong Nguyen, Pascal Crépey, Ronan Garlantezec, Véronique Doré, Audrey Dumas, Mohamed Ben Mechlia, Pierre Tattevin, Jean Gaudart, Bruno Spire, France Lert, Yazdan Yazdanpanah, Constance Delaugerre, Marion Noret, Jeremy Zeggagh

**Affiliations:** ^1^Service de Maladies Infectieuses et Tropicales, Hôpital Avicenne, AP-HP, Bobigny, France; ^2^LEPS Laboratoire Éducations et Pratiques de Santé, Université Paris 13, Bobigny, France; ^3^CIC Cochin Pasteur, AP-HP, Paris, France; ^4^Univ Rennes, EHESP, CNRS, INSERM, Arènes - UMR 6051, RSMS - U 1309, Rennes, France; ^5^CHU de Rennes, University Rennes, INSERM, EHESP, Irset (Institut de Recherche en Santé, Environnement et Travail) – UMR_S 1085, Rennes, France; ^6^ANRS, Agence Nationale Recherche Sida, Paris, France; ^7^Infectious Diseases and Intensive Care Unit, Pontchaillou University Hospital, Rennes, France; ^8^Aix Marseille University, APHM, INSERM, IRD, SESSTIM, ISSPAM, UMR1252, Hop Timone, BioSTIC, Biostatistic and ICT, Marseille, France; ^9^Aix Marseille University, APHM, INSERM, IRD, SESSTIM, ISSPAM, UMR1252, Marseille, France; ^10^Service de Maladies Infectieuses et Tropicales, Hôpital Bichat, AP-HP, Paris, France; ^11^Service de Virologie, Hôpital Saint-Louis, AP-HP, INSERM U944, Université de Paris, Paris, France; ^12^Renarci, Centre Hospitalier, Annecy, France; ^13^Service de Maladies Infectieuses et Tropicales, Hôpital Saint-Louis, AP-HP, Paris, France

**Keywords:** SARS-CoV-2, respiratory virus, nightclub, respiratory virus transmission, vaccine

## Abstract

**Introduction:**

The SARS-CoV-2 pandemic led to the implementation of several non-pharmaceutical interventions (NPIs), from closings of bars and restaurants to curfews and lockdowns. Vaccination campaigns started hoping it could efficiently alleviate NPI. The primary objective of the “Indoor Transmission of COVID-19” (ITOC) study is to determine among a fully vaccinated population the relative risk of SARS-CoV-2 transmission during one indoor clubbing event. Secondary objectives are to assess the transmission of other respiratory viruses, risk exposure, and attitudes toward COVID-19 vaccination, health pass, and psychological impact of indoor club closing.

**Methods and analysis:**

Four thousand four hundred healthy volunteers aged 18–49 years and fully vaccinated will be included in Paris region. The intervention is an 8-hour indoor clubbing event with no masks, no social distance, maximum room capacity, and ventilation. A reservation group of up to 10 people will recruit participants, who will be randomized 1:1 to either the experimental group (2,200 volunteers in two venues with capacities of 1,000 people each) or the control group (2,200 volunteers asked not to go to the club). All participants will provide a salivary sample on the day of the experiment and 7 days later. They also will answer several questionnaires. Virological analyses include polymerase chain reaction (PCR) of salivary samples and air of the venue, investigating SARS-CoV-2 and 18 respiratory viruses.

**Ethics and dissemination:**

Ethical clearance was first obtained in France from the institutional review board (Comité de Protection des Personnes Ile de France VII - CPP), and the trial received clearance from the French National Agency for Medicines and Health Products (Agence National de Sécurité du Médicament - ANSM). The trial is supported and approved by The Agence Nationale Recherche sur le SIDA, les hépatites et maladies émergences (ANRS-MIE). Positive, negative, and inconclusive results will be published in peer-reviewed scientific journals.

**Trial registration number:**

IDR-CB 2021-A01473-38. Clinicaltrial.gov, identifier: NCT05311865.

## Key points

Study of the transmission of SARS-CoV-2 in vaccinated persons in real life conditions participating in a nightclub.

## Introduction

### Transmission of respiratory viruses, including COVID-19

The SARS-CoV-2 is transmitted through virus-containing droplets (> 5 to 10 μm) and aerosols ( ≤ 5 μm) exhaled from infected individuals during breathing, speaking, coughing, and sneezing (1). It can be transmitted by asymptomatic infected individuals and during the incubation period (2). Superspreading events have been identified mainly in closed spaces, without wearing a mask with no test condition to enter (3). Moreover, the indoor transmission increases with the number of infected participants' time of exposure and lack of ventilation. The risk of transmission varies widely depending on the setting and activity (singing, eating…etc.), and has been originally reported to ranging from 10.3% in a train (4) to 78% in a church for the pre-alpha variant (5, 6). Seasonal respiratory viruses, such as influenza, respiratory syncytial viruses (RSVs), metapneumovirus, rhinovirus, adenovirus, bocavirus, adenovirus, and coronaviruses (NL63, HKU1, OC43, and 229E) are a concern because they can cause a heavy burden especially in new-born, elderly, immunocompromised patients, or with comorbidities. As for SARS-CoV-2 (7–10), these viruses are transmitted *via* droplets and aerosols, depending on temperature and humidity, following a “U” shape curve (10). However, there is a lack of data regarding their spread in indoor settings (11).

### The vaccine against COVID-19 and its efficacy in transmission for pre-alpha and alpha variant

COVID-19 vaccines have been shown to be highly effective (>90%) in preventing SARS-CoV-2 infections in pre-alpha and alpha variants, according to the data collected in the USA, UK, Israel, and Qatar. Moreover, observational data have also demonstrated their efficacy to prevent the disease, especially in healthcare and household settings (40–50%) (12).

### The vaccine against COVID-19 and its efficacy on the delta variant and its transmission

The approved COVID-19 vaccines have lower effectiveness against mild forms of SARS-CoV-2 infections but remain effective to prevent hospitalizations (13–15). Contrasting results have been published regarding the transmission of SARS-CoV-2 among vaccinated. Evidence of the COVID-19 outbreak in a large, vaccinated population have been reported in Provincetown (USA) (16), which has also shown a superspreading event, while transmission in a healthcare setting occurred in Vietnam (17). However, another study in the USA showed few work-related secondary cases in vaccinated healthcare workers. A recent prospective cohort study in the UK found a modest protective effect on COVID-19 transmission (18): The secondary attack rate (SAR) in household contacts exposed to the delta variant was 25 and 38% in vaccinated and unvaccinated contacts, respectively. SAR among household contacts exposed to fully vaccinated index cases (25%; 95% CI 15–35) was similar to household contacts exposed to unvaccinated index cases (23%; 15–31).

### Transmission of COVID-19 in indoor clubbing

Indoor clubbing events may pose a significant risk of SARS-CoV-2 transmission: there is no physical distancing between individuals when operating at full capacity. A high density of persons and interactions between individuals include high-risk behavior such as singing, dancing, or physical contact (19). In addition, without proper air filtration or air renewal systems, infectious aerosols may accumulate and lead to a superspreading event. In an unvaccinated population, these places can be at high-risk place of SARS-CoV-2 transmission (20). Few studies have looked at the transmission of SARS-CoV-2 in festive events but none in vaccinated people (15, 21–23).

### Rationale, knowledge gap, and issues

More research is needed to measure the transmission of the delta variant during indoor live events: current evidence shows that vaccines are less effective in preventing transmissions, depending on the context. Moreover, we need to study transmission in a real-life setting where tests will no longer be needed to participate in an event, because of cost and practical issues.

### Hypothesis and objectives

#### Hypothesis

Our study hypothesis is that the participation in an indoor clubbing event is not associated with an increased risk of transmission of SARS-CoV-2 in fully vaccinated participants, while other respiratory viruses' transmission is increased.

#### Objective

The primary objective is to assess the absence of an increase in the risk of SARS-CoV-2 infection 7 days after an indoor club event in the group participating in the event, being fully vaccinated and without a face mask, compared to a non-participating group.

## Methods and analysis

### Study design

The present protocol is designed according to the CONSORT statement for randomized controlled trials (24) and the complete version has been registered under the number “021-A01473-38” with the French research authorities. Randomized, open-label, non-inferiority interventional cluster study with 4,200 participants divided 1:1 into two groups of volunteers recruited *via* social networks and *via* nightlife professional union networks.

Participants must have the same types of social interactions that they might have outside of the study. Thus, randomizing participants individually would result in splitting groups of participants who planned to spend the evening together. Participants who are “alone” at a party would not behave as they would under real-life conditions with friends. On the online booking module, participants will be able to mention the email of the people with whom they want to participate in the experimentation.

Participants will be allocated by reservation groups of up to 10 people through a central electronic service (WEEZEVENT, (25)).

Participants will be randomized into two arms:

- Experimental group: Indoor clubbing event
- Control group: No participation in the indoor clubbing event

### Study population

Eligibility criteria are designed to select a sample from people attending parties, all in real-life conditions. The study focuses on individuals aged from 18 to 49 years without comorbidities to further reduce the risk of potential complications and transmission to people at risk.

#### Inclusion criteria

Patients must fulfill the following criteria prior to trial enrollment:

- Aged between 18 and 49 years old included without comorbidity
- At inclusion, completed and checked vaccination defined at that time as:

° Two doses of vaccine (Vaccines Pfizer BioNTech^Ⓡ^/AstraZeneca^Ⓡ^/Moderna^Ⓡ^) at 4 weeks intervals plus 7 days.
° Or one dose of Vaccine Janssen plus 4 weeks
° Or in a patient with positive serology for SARS-CoV-2 (SARS-CoV-2 specific Ig G anti S and Ig anti N, by the enzyme-linked immunosorbent assay (ELISA) test, no rapid test accepted) or a history of proven infection: one dose of any COVID-19 vaccine plus 7 days

- People who declared to have no risk factor for severe COVID-19 according to the French Health High Authority (*Haute Autorité de Santé - HAS* (26))
- People who declared not to live in the same place as someone with these risk factors
- People residing in Paris region
- People affiliated with the National health insurance (*Sécurité sociale*)

#### Non-inclusion criteria

Patients with any of the following criteria are not eligible for trial enrollment:

- Presence of symptoms of COVID-19 in the 2 weeks preceding the event
- Pregnant woman or woman who declares not having an effective contraception method
- Self-identification of medical conditions or comorbidities identified as a risk factor for severe COVID-19
- People living with a person with these risk factors
- Confirmed diagnosis of SARS-CoV-2 within 2 weeks before the event
- Participants under tutorship or curatorship
- Participants unable to give free and informed consent.

### Randomization

The randomization list will be centralized and computer-generated using permuted blocks of varying lengths. Patients will be randomly allocated to study arms by means of the central 24–7 Internet randomization service provider (KapCode^®^). The framework will be secured by a login and a password.

### Participant timeline

A communication campaign to recruit participants will be deployed *via* the social networks of partner artists and nightclubs. Posters and flyers will be distributed in five places in the capital.

Before inclusion, the participants will be able to pre-register for the event *via* a dedicated website https://revienslanuit.org/.

Participants will be asked to complete an initial inclusion questionnaire on the website.

Three days before the event (from D-4 to D-2), participants will have to register for an inclusion visit where they will have to pick up their reverse transcriptase-polymerase chain reaction (RT-PCR) SARS-CoV-2 saliva kits.

Two kits containing one tube to collect saliva samples (for D0 + D7) with prepaid envelopes will be given to all participants at this visit. An email address will be given to participants to inform investigators in case of bad realization of the test to get a new sampling kit. It will be explained to perform the test properly in case of symptoms.

It is during the inclusion visit that participants will have to show their up-to-date vaccination certificates.

All vaccinated participants will be included and randomized.

The day before the event, only participants in the experimental group will receive an email with a ticket for the event. For the control group, individuals are asked to respect current health measures and not to go to discotheques on the experimentation day. To facilitate their adherence to the protocol, they will receive compensation in the form of a gift voucher, given at the end of the last questionnaire.

- On Day 0, people randomized in the experimental group will come to the venue with their ticket and their saliva sample to participate in the event. At the entrance, they will have to show their certificate of vaccination and their ticket to the staff members. Individuals randomized in the control group would not be able to attend the event but they will have to send their saliva samples by mail.
- On Day 1, participants randomized to the experimental arm of the study will receive a questionnaire to follow-up within 24 h after the party and those in the control group will receive a questionnaire to assess whether they also attended a party on the same day.
- From Day 3 to Day 10, all participants will be monitored regularly *via* a short questionnaire to collect information on their health status and the possible appearance of symptoms suggestive of COVID-19. Email and SMS reminders will be sent in case of non-response.
- On Day 7 (±1 day), all the participants (experimental group and control group) will have to send their D7 saliva sampling kit *via* mail. Upon receipt of this tube, the participants in the control group will receive compensation in the form of a gift certificate. In case of a positive test, participants will be contacted individually by the investigators' physician for contact tracing in the context of the research.

### Location of the study and event

The 2,200 participants of the experimental group will be randomly assigned to one of the two indoor venues called “La Machine du Moulin Rouge” or “La Bellevilloise.” The reservation group of participants will be assigned to the same venue to respect the randomization by block.

**Figure 1:**
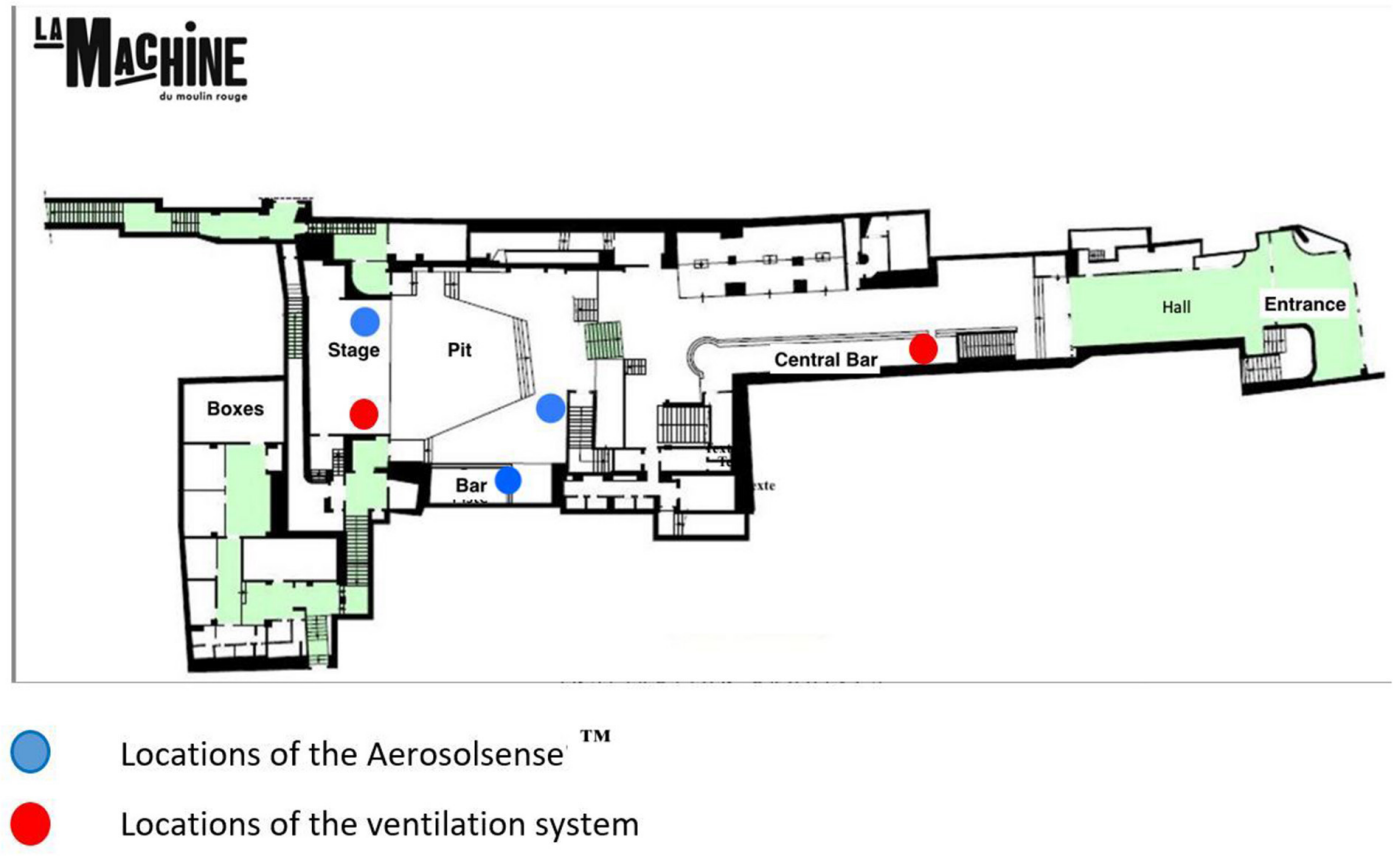
Plan of the room “La Machine du moulin rouge” and location of the AerosolSense™ and ventilation.

**Figure 2:**
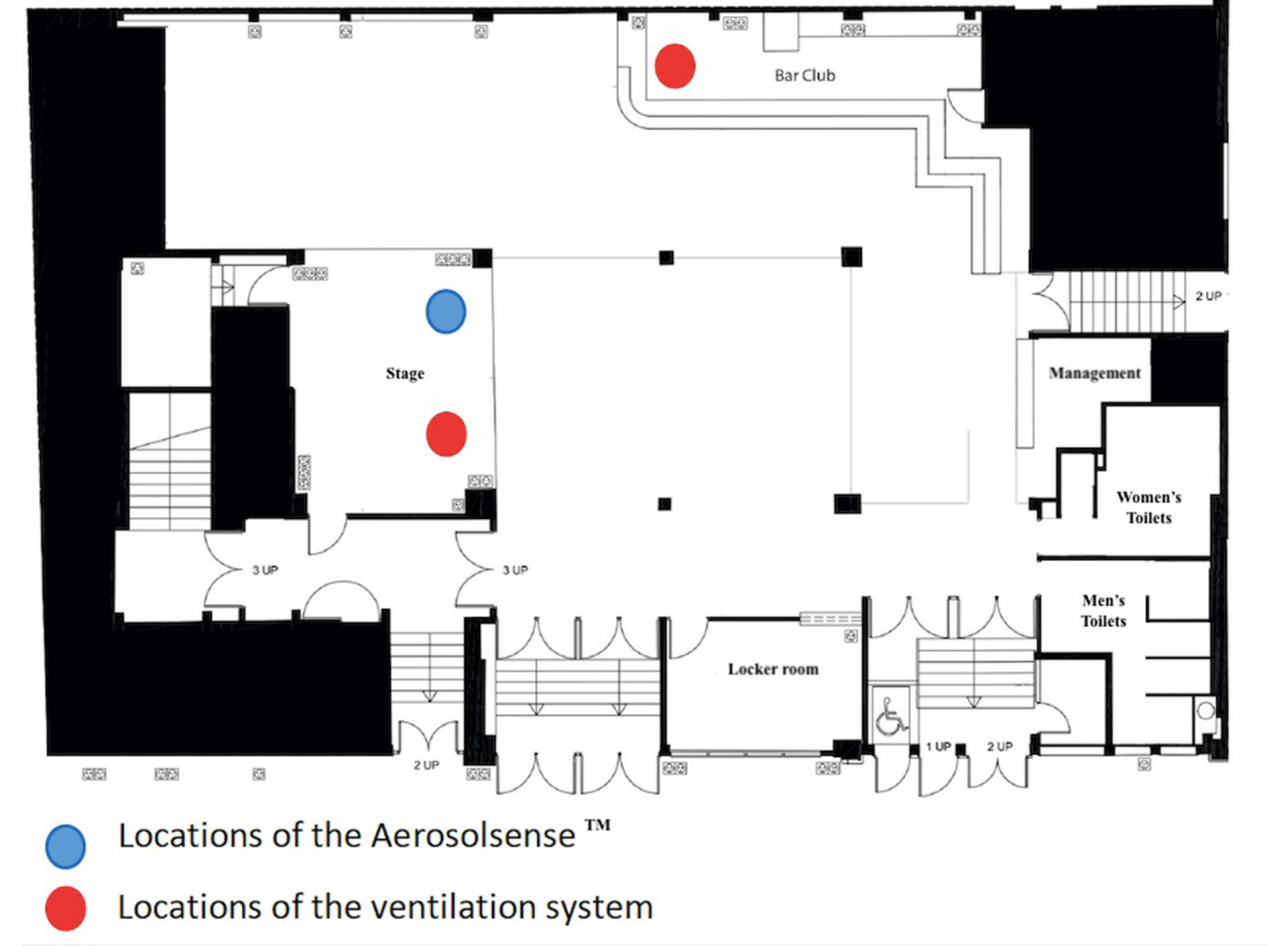
Plan of the room “La Bellevilloise” and locations of the AerosolSense™ and ventilation.

“Machine du Moulin Rouge” is a venue with a surface area of 500 m^2^. It has two bars and two ventilation systems: one at the central bar whose ventilation capacity is 10,627 m^3^/h, and another on the main stage whose capacity is 11,000 m^3^/h (Figure 1). “Bellevilloise” is a venue with a surface area of 450 m^2^ and has only one bar. Its ventilation system operates at 7,500 m^3^/h thanks to two machines located at the dance floor and bar levels (Figure 2).

The doors will be open from 11 to 6 pm and participants can arrive at any time. The music program will be composed of eight disk jockeys (DJs) distributed over the two venues. The timetable and distribution of DJs in each venue will not be known to the participants to avoid selection bias. The ventilation systems will be started 3 h before the event. During the experiment, masks are not mandatory except for staff and no social distancing measures will be required. The hydro-alcoholic gel will be made available at various locations.

Three AerosolSense™ systems (Thermo Fisher) at “Machine du Moulin Rouge” and one at “Bellevilloise” will be installed to detect viruses in the air. The system will be used to determine viral contamination in the 12 h prior to the start of the event, during the entire event (11 pm−6 am), and for 12 h after the event.

The cartridges of each instrument used for each period will be tested using a multiplex PCR detecting a panel of respiratory viruses, including SARS-CoV-2 (Filmarray Respiratory Panel 2.1, bioMérieux^®^). For SARS-CoV-2 positive samples, semi-quantitative estimations will be performed using real-time RT-PCR technique with the determination of the cycle threshold (Ct).

### Primary endpoint

The primary endpoint is the number of participants in each group with a positive salivary SARS-CoV-2 RT-PCR on day 7 after the date of the event.

### Secondary endpoints

Secondary endpoints are:

1. Numbers of asymptomatic, symptomatic, and severe SARS-CoV-2 infections according to a self-assessment by a questionnaire sent online from D3 to D10 after the date of the event, and medical follow-up.
2. Whole-genome sequencing of SARS-CoV-2 detected in participants' samples.
3. Comparison of the sequences of the complete SARS-CoV-2 genomes in the participants with a positive test on D7.
4. Detection of seasonal respiratory viruses including SARS-CoV-2 in the air, by the AerosolSense™ system (Thermo Fisher Scientific).
5. Assessment of acceptability to download and use the recommended tracking application (“TOUSANTICOVID”) through surveys sent online after the party (eCRF) (27).
6. Adherence to the health protocol by a questionnaire sent online to participants and organizers after the evenings.
7. Online questionnaire on motivation and barriers to COVID-19 vaccine and *COVID Certificate* (Digital or paper presentation of health proof, according to the regulations in force).
8. Survey of close contact behavior during the event.
9. Number of participants in each group with a positive seasonal respiratory viruses PCR on day 7 after the date of the event.
10. Numbers of asymptomatic, symptomatic, and severe seasonal respiratory virus infections according to a self-assessment by a questionnaire sent online from D3 to D10 after the date of the event, and medical follow-up.
11. Detection of seasonal respiratory viruses in the participant's saliva at D0/D7 by RespiFinder^®^ 2Smart assay (PathoFinder).

### Data collection

The trial will be conducted in accordance with relevant regulations and standard operating procedures, including data protection. The data will be collected on an electronic case report form. All necessary precautions to ensure the confidentiality of information regarding investigational medicinal products, trial participants, and in particular the identity of the participants and the results obtained will be taken.

### Statistical considerations

#### Sample size computation

In a non-inferiority design, the sample size depends on the probability of the observed event (here a SARS-CoV-2 infection), the power of the study, and a threshold of clinical significance.

To define this threshold, we consider that the exposure of a vaccinated population to the event has to lead to an equal or less number of secondary infections than in a non-exposed unvaccinated population. However, according to the risk/benefit balance and the random assignment, both our control and intervention arms need to be fully vaccinated. Consequently, we need to estimate the level of infection which could have been observed in the control group if it had not been vaccinated. Assuming a vaccine efficacy against infection of 80% would mean that the risk of infection is divided by 5, hence we consider that the incidence observed in the control group could have been multiplied by 5 if the control group was not vaccinated. Consequently, we set the non-inferiority threshold to five times the incidence observed in the control group.

**Figure 3:**
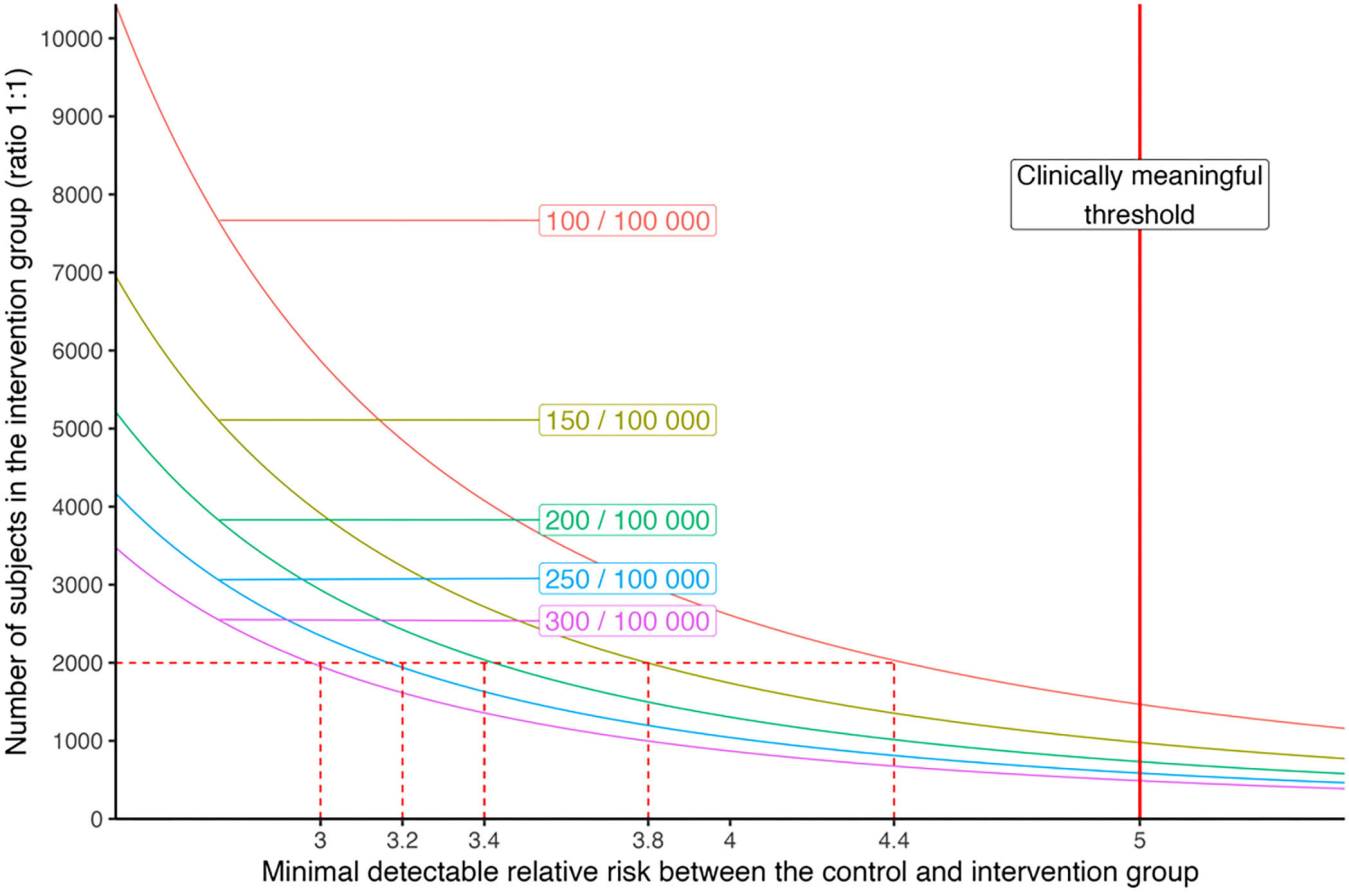
Minimal detectable relative risk depending on the number of subjects in the intervention group for different levels of incidence of SARS-CoV-2 infection detected in the general population. With 2,000 subjects in each arm (intervention and control), we have a power of 80% to detect a relative risk of 3–4.4 depending on the level of SARS-CoV-2 incidence.

As shown in Figure 3, the additional risk could be detected as a function of incidence and sample size, with 80% power and alpha risk of 5%. We corrected observed incidence rates in the population to account for 38% under-detection and 80% vaccine protective effect, as observed in France in 2020 (28).

For incidences in the general population of between 100 and 300 positive tests per week per 100,000 people, the detectable excess risk would be between 4.4 and 3 (respectively) for 2,000 participants in the intervention group and 2,000 participants in the control group (ratio 1:1; Figure 3). We simulated the impact of the clustering effect, considering an increased risk of infection within groups of participants who came together to the event. Considering that the secondary attack rate within those groups would be decreased by 80% by the vaccine protection, we could assume that the intra-cluster correlation would be negligible and that the design effect would be close to 1. With a planned attrition rate of 10% of participants for the primary outcome on D7, we plan to randomize a total of 2,200 participants in the “experimental” arm and 2,200 in the “control” arm.

#### Analysis of the primary endpoint

The primary endpoint of the current study is positive SARS-CoV-2 proportion in saliva samples 7 days after the event in the experimental group compared to the non-experimental groups. Statistical analyses were performed on an intention-to-treat basis.

#### Analysis of secondary endpoints

- Risk factors of SARS-CoV-2 contamination (as defined as positive RT-PCR): we will use multi-level regressions to account for the cluster effect. Variables in the model will include socio-demographic data, size of the reservation group, time spent in the event, and use of transportation
- By adherence to the health protocol evaluated by online questionnaire, measure quantity, and length of individual contacts (with and without mask) between participants: This analysis will help to identify the time and location of exposure, to make recommendations during the event. This will also feed agent-based transmission models.
- Identify superspreading events: every positive result will lead to contact tracing to identify transmission paths.
- Study adherence to sanitary protocol, to identify a way to improve.

### Committees for the research

The ITOC scientific committee has developed and implemented the protocol in France. It ensures that the trial is conducted in accordance with ethical principles and respects participants' safety, takes any decision on any changes made to the design of the ITOC trial, and on the reporting of the trial results, including regarding the publication policy. The study was registered on *clinicaltrials.gov* under the ID: NCT05311865. The protocol described in this article is the V 6.0 of the ITOC protocol.

### Patient and public involvement

No patient was involved in the design or implementation of this study. Focus groups with potential participants were conducted to determine how best to compensate the control group participants and the best communication elements.

## Discussion

We describe here the protocol of a trial design on SARS-CoV-2 transmission during an indoor clubbing event among the fully vaccinated population. Our study will be able to determine the risk of Delta variant SARS-CoV-2 and other respiratory viruses' transmission in the highest-risk context. There have been several experiments conducted to assess the transmission of COVID-19 during indoor events (Table 1).

**Table 1:** Follow-up timeline for research participants.

**Timepoint (day)**	**Study period**						
	**Enrolment**		**Post-allocation**				**Close-out**
	**–D3 to D−1**	**DO**	**D3 (+/−1)**	**D7 (+/−1)**	**D10 (+/−1)**	**D30 (+/−1)**	**D30**
**Enrolment**							
Information	X						
Consent	X						
Eligibility questionnaire	X						
**Interventions**							
Randomisation		X					
Experimentation in the venue		X					
Not to go to club		X					
**Assessments**							
PCR Saliva		X		X			
Post experimentation questionnaires			X				
Follow-up symptoms questionnaire			X	X	X		
Follow-up symptoms questionnaire COVID patients			X	X	X	X	

The “Risk Prediction of Indoor Sports and Culture Events for the Transmission of COVID-19” (RESTART-19) study, in August 2020 in Germany, aimed to map the behavior of participants during a live, indoor concert (split into two parts with a break) (15). It recruited 1,212 healthy volunteers aged 18–50, with a negative PCR test on throat swabs 2 days before the event, with no fever, and wearing an N95 mask on the day of the event. Three configurations have been tested with different social distancing modalities. They detected several contacts at the entrance and during the break, which were less than 15 min, and identified the efficacy of social distancing and the importance of ventilation (15). In March 2021, Llibre et al. performed a large-scale screening study of 5,000 participants in a live indoor concert with masks and no social distancing (29). They performed same-day antigen-based rapid diagnostic tests (Ag-RDTs) and a post-event follow-up *via* electronic health records or phone calls, in collaboration with the Catalan Public Health Department, which provides set up a centralized epidemiologic surveillance system for polymerase chain reaction (PCR). Six participants, not vaccinated, were tested positive, of whom three have been infected by someone who did not attend the concert. There was no control group. In the UK, the government has planned for ≪ Events Research Programme ≫, which includes nine studies to study the risk of transmission in various contexts. Among these, two clubbing events were organized in April 2021, with more than 6,000 participants. Participants had to present negative Ag-RDTs to participate and were invited to perform another Ag-RDT 7 days after the event. No evidence for increased risk of transmission was found, but less than 10% of participants have provided results before and after the event (23). More recently, another experiment (Clubculture reboot) has been organized in August 2021, with more than 2,000 participants in clubbing events without masks or social distancing, with adults who had nasopharyngeal PCR before and 1 week after. According to the organizer, no new infection has been detected (22). These results must be balanced with another study in Catalonia, which found a higher SARS-CoV-2 transmission among participants of mass gathering events. However, there was no control group (30).

Only two studies were randomized with a control group. The PRIMA-CoV study in Spain is a randomized controlled open-label trial to assess the effectiveness of a comprehensive preventive intervention for a mass gathering live indoor concert based on systematic same-day screening by PCR on a nasopharyngeal swab for all participants. This study has randomized 1,140 people for an event in December 2020. At baseline, 3% of participants in each arm were positive. One week later, no evidence of increased incidence in the experimental group was found with Ag-RDTs, use of masks, and adequate air ventilation (31). In France, the SPRING study is a prospective, randomized controlled trial on the transmission of SARS-CoV-2 during a live concert event, with a medical mask and adequate ventilation. Incidence was measured by PCR on salivary samples, on the day of the event and 7 days after. No difference was found between the experimental and the control arm: eight participants were tested positive among 3,917 participants in the intervention arm (incidence [CI 95%]: 0.20% [0.09; 0.40]), compared with three among 1,947 participants in the control arm (incidence: 0.15% [0.03; 0.45]) confirming the non-inferiority. Incidence rates were similar to the observed age-standardized 14-day incidence rate locally (21).

## Strengths and limitations

All these studies, except the mass gathering events in Borriana (Spain) (30), did not show an increased risk of transmission during live indoor events. However, every participant had to present a recent negative test (Ag-RDT or PCR), which would be difficult to implement in real life where tests are expensive, and with limited access. Moreover, these studies occurred during pre-alpha or alpha variant circulation. To our knowledge, this is the first protocol in which the result of the SARS-CoV-2 test does not condition entry to the event under real-life conditions in vaccinated participants during periods of delta virus circulation. The ventilation conditions of the rooms are precisely detailed in this protocol. Finally, our study will use analyses from the air. The AerosolSense™ system is a reliable system that has been used to evaluate the detection of SARS-CoV-2 in rooms of patients with COVID-19 (11).

Another strength of our study is to extend to other respiratory viruses, that share transmission similarities with SARS-CoV-2. We will be able to compare the transmissions, which may allow us to estimate the protection afforded by COVID-19 vaccines.

Our study faced many challenges. An important was organizational: we had to find the right balance between finding the right timing with an appealing line-up of popular artists to motivate participants to enroll while maintaining the best clinical trials standards. We had to reassess regularly the feasibility of the study depending on changing regulation and to discuss equipoise regarding the effectiveness of COVID-19 vaccines on delta variant in severe cases. Therefore, the power of the study is limited by the capacity of finding adequate venues, COVID-19 incidence, and recruitment capacity and adherence, which has proven to be challenging in previous studies. We also assumed that participants in the control arm might not stay at home. To address these issues, we conducted a focus group to explore different communications slogans and the best compensation for the control group. Moreover, we designed surveys to report different high-risk behaviors that may occur during the day of the event in the control group.

These real-life interventional epidemiological studies provide new perspectives and opportunities for research on the transmission of SARS-CoV-2 as well as other types of research that evolve with changes in society (32, 33).

### Strengths and limitations of this study

- Innovative design to investigate the transmission of COVID-19 and other respiratory viruses.
- Healthy vaccinated participants experiencing a clubbing party in real-life conditions without a mask and in full capacity.
- Randomized controlled clinical trial in compliance with good clinical practice.
- Risk of non-compliance in the control group.
- Power calculation performed several weeks before intervention is based on SARS-CoV-2 incidence.

## Dissemination

Results will be communicated at scientific meetings and submitted for publication in peer-reviewed journals. According to the information sheet, participants will be informed of the overall results at the end of the trial. In addition, participants are informed of the discontinuation of a treatment arm in the trial after validation by the ethics committee.

## Trial status

This trial has begun on 13 October 2021. To date, 1,216 patients have been included.

## Ethics statement

The studies involving human participants were reviewed and approved by the Ethics and dissemination: Ethical clearance was first obtained in France from the institutional review board (Comité de Protection des Personnes Ile de France VII - CPP), and the trial received clearance by the French National Agency for Medicines and Health Products (Agence National de Sécurité du Médicament - ANSM). The trial is supported and approved by the Agence Nationale Recherche sur le SIDA, les hépatites et maladies émergences (ANRS-MIE). Positive, negative and any inconclusive results will be published in peer-reviewed scientific journals. Trial registration number: Eudra-CT 2021-A01473-38. Clinicaltrial.gov ID: NCT05311865. The patients/participants provided their written informed consent to participate in this study.

## Author contributions

JZ, LL, and JG: conceptualization and investigation. GR: data curation. PC: methodology. CD and YY: supervision. MN, JZ, LL, and JG: writing original draft. CD, YY, and PC: writing review and editing. All authors have read and approved the final manuscript.

## Funding

The trial is funded by a grant from the French Ministry of Health. The trial is promoted by Agence Nationale de Recherche sur le SIDA et les Hépatites et Maladies Infectieuses Emergentes (ANRS-MIE, France). Award/Grant Number: N/A.

## Conflict of interest

The authors declare that the research was conducted in the absence of any commercial or financial relationships that could be construed as a potential conflict of interest.

## Publisher's note

All claims expressed in this article are solely those of the authors and do not necessarily represent those of their affiliated organizations, or those of the publisher, the editors and the reviewers. Any product that may be evaluated in this article, or claim that may be made by its manufacturer, is not guaranteed or endorsed by the publisher.
